# The Mediating Role of Dietary Inflammatory Index in the Association between Eating Breakfast and Obesity: A Cross-Sectional Study

**DOI:** 10.3390/nu14204378

**Published:** 2022-10-19

**Authors:** Mengzi Sun, Xuhan Wang, Ling Wang, Wenyu Hu, Yixue Yang, Nan Yao, Jing Li, Zechun Xie, Ruirui Guo, Yuxiang Wang, Bo Li

**Affiliations:** Department of Epidemiology and Biostatistics, School of Public Health, Jilin University, Changchun 130021, China

**Keywords:** dietary inflammatory index, eating breakfast, obesity

## Abstract

Obesity is closely related with diet, including the regularity of meals and inflammation in the diet. No previous study focused on the associations among eating breakfast, which is regarded the most important meal, dietary inflammation, and obesity. This study analyzed data from the National Health and Nutrition Examination Survey (NHANES) from 2007 to 2018, with 23,758 participants involved. Obesity and dietary inflammation were measured by body mass index (BMI) and dietary inflammatory index (DII), respectively. Eating breakfast was defined by two days of dietary recalls based on NHANES dietary data. Pro-inflammatory diet and skipping breakfast were positively associated with obesity in the whole population. Compared with eating breakfast in both recalls, skipping breakfast had the higher OR of obesity, especially for individuals who reported no recall. Participants with diabetes were the sensitive population of these associations. Compared with participants who reported breakfast in both recalls, the mediated proportion of participants reported breakfast in one recall and in no recall were 24.71% and 27.34%, respectively. The association between eating breakfast and obesity was partly mediated by DII. We recommended eating breakfast regularly to reduce dietary inflammation, as well as further obesity, especially for diabetic populations.

## 1. Introduction

Recent statistics showed that overweight and obesity have continued to rise globally, with more than 2 billion overweight people accounting for approximately 30% of the world’s population [1]. Obesity has been determined to be associated with many adverse outcomes, including asthma, nonalcoholic fatty liver disease (NAFLD), and female subfertility [2,3,4]. A report that covered 195 countries over 25 years indicated that nearly 70% of deaths related to high BMI were due to cardiovascular disease and over 60% of those deaths occurs among the obese [5].

Breakfast has been described as the most important meal of the day, and it provides nutrients for the body after overnight fasting [6]. Breakfast has been implicated in weight control, cardio-metabolic risk factors, and cognitive performance in recent years, although the literature remains inconclusive as to the precise health benefits of breakfast [7]. Cross-sectional and prospective observational studies have overwhelmingly found that skipping breakfast was directly associated with overweight and obesity [8]. Skipping breakfast may disrupt circadian rhythms [9] and may lead to metabolic alterations, including the dysregulation of circulating satiety hormones [10]. While the mechanisms connecting breakfast consumption and chronic disease risk have not been fully understood, increased inflammation in breakfast skippers has been suggested as one potential pathway connecting these associations [11,12].

Dietary Inflammatory Index (DII) was a literature-derived score developed to evaluate the inflammatory potential of the diet and link diet to inflammation [13]. Meanwhile, DII could perform a quantitative measure for assessing the relationships between diet and health outcomes [14]. Most studies found that DII was associated with an increased risk of obesity, T2DM, and CVD with some relationships being sex-specific [15]. A previous study focused on the children reported that breakfast skipping was associated with higher Children’s Dietary Inflammatory Index (C-DII) [16]. Moreover, another study also found that proportion of high DII of individuals who always consumed breakfast was lower than individuals who seldom or sometimes consumed breakfast [17].

However, there were few studies focused on the association among eating breakfast, DII, and obesity. Therefore, we hypothesized that the association between eating breakfast and obesity was mediated by DII and aimed to quantify the effect of eating breakfast and DII on obesity based on the National Health and Nutrition Examination Surveys (NHANES). Furthermore, our results might provide a reference for specifying prevention measurements of obesity in eating breakfast.

## 2. Methods

### 2.1. Sample

The NHANES, which were conducted by Centers for Disease Control and Prevention (CDC), aimed to assess the health status of the U.S. non-institutionalized civilian population. This survey utilized a complex probability sampling design and collected information by standardized interviews, physical examinations, and tests of biological samples [18]. A total of 34,770 adults aged over 20 years old were enrolled in NHANES in 2007–2018. We excluded 372 participants who were pregnant and lactated, 228 participants who had abnormal energy (total energy intakes of <500 or >5000 kcal/day for females and <500 or >8000 kcal/day for males), 7803 participants had missing nutrition data, 271 participants had missing BMI data, 2242 participants had missing income data, 96 participants had missing other covariable data. Finally, 23,758 participants were involved in this study. Furthermore, 3283 participants with diabetes were excluded, 20,475 participants were involved in a follow-up sensitivity analysis. The detailed situation was shown in Figure S1 (Supplementary Materials). Moreover, a total of 8150 elderly were involved in the sub-groups’ analysis.

### 2.2. Definition of Eating Breakfast

Each NHANES recall collected information on the name and clock time of each meal event in the report. All recalled food or beverage items reported within a clock time were given the same dietary event designation. We considered participants who mentioned breakfast, desayuno, or almuerzo as breakfast reporters. If only boiled water was mentioned for breakfast, we did not consider it breakfast [19].

### 2.3. Definition of Dietary Inflammatory Index (DII)

We used the revised version of the DII calculation that was developed by Shivappa et al., and the specific algorithm was detailed in a previous study [1]. In this study, 27 nutrients were used for the calculation of the DII, which included alcohol, vitamin B12/B6, β-carotene, caffeine, carbohydrate, cholesterol, total fat, fiber, folic acid, Fe, Mg, Zn, Se, MUFA, niacin, n-3 fatty acids, n-6 fatty acids, protein, PUFA, riboflavin, saturated fat, thiamin, vitamins A/C/D/E. Importantly, even if the nutrients applied for the calculation of DII are less than 30, the DII scores are still available [13]. Participants were divided into anti-inflammatory diet (DII < 0) and pro-inflammatory diet (DII ≥ 0).

### 2.4. Definition of Other Variables

Body mass index (BMI) was divided into three categories: underweight or healthy weight (BMI < 25.0 kg/m^2^), overweight (25.0 kg/m^2^ ≤ BMI < 30.0 kg/m^2^), or obese (BMI ≥ 30.0 kg/m^2^) [20].

Smoking status was divided into three categories: Non-smokers were defined as those who never had at least 100 cigarettes in their lifetime; former smokers were defined as those who had at least 100 cigarettes but did not smoke now; and current smokers were defined as participants who had at least 100 cigarettes and reported a number of cigarettes per day in the past 30 days [21]. Physical activity was assessed with a physical activity questionnaire. Metabolic equivalent (MET) was measured according to how long and hard every participant worked out. Active physical activity was defined as or more than 599 MET, or more than 149 min of moderate physical activity, or more than 74 min of vigorous physical activity [22]. Diabetes was defined as any of the following: glycosylated hemoglobin (HbA1c) ≥ 6.5%, fasting blood glucose ≥ 126 mg/dL, or current use of insulin [23].

### 2.5. Statistical Analysis

Mean and standard error (SE) were used to describe the continues variables, and *t*-test was performed for the comparison. Unweighted frequency and weighted percentage were used to describe the categorical variables, and chi-square test was conducted for the comparison. Binary logistic regression was used to analyze the association between dietary inflammation and obesity under adjustments. All the description and regression analyses were performed under the complex sampling weight of NHANES.

**Figure 1:**
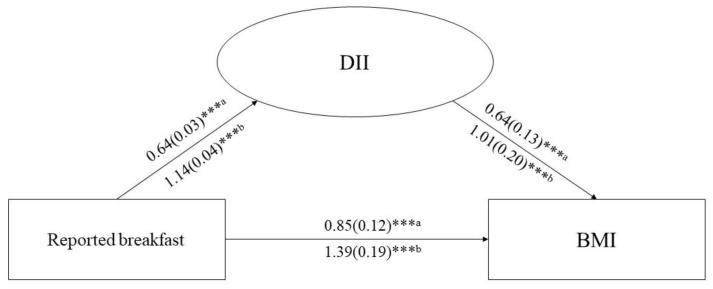
Regression results of the mediation analysis models. Notes: Exposure: Reported breakfast; Outcome: BMI; Mediator: DII. Model adjusted for Sex, Age, Race, Education status, Marital status, Income status, Smoking status, Physical activity, Diabetes, and Energy intake. The regression coefficient (standard error) is shown on the paths. *** *p* < 0.001. ^a^ Reported breakfast in one recall. ^b^ Reported breakfast in no recalls.

Then, to investigate whether the DII levels mediated the association between eating breakfast and BMI, three pathways (a, b, and c) were used to assess the mediation (Figure 1). Total effect evaluated the association between eating breakfast (exposure) and BMI (outcome). Path a assessed the association between eating breakfast and DII (mediator). Path b measured the association between DII (mediator) and BMI (outcome). The influence of DII on the link between eating breakfast and BMI was assessed through path c (direct effect). The proportion of the mediated effect was calculated using the following formula: (mediated effect/total effect) × 100%. Bootstrapping was used for significance testing for the mediation analysis.

To validate the robustness of the associations of eating breakfast with dietary inflammation and obesity, sensitivity analysis was performed via stratified logistic analysis, and mediation analysis was then performed after excluding the specific population from the stratified logistic analysis. All statistical analyses were conducted by IBM SPSS 26.0 and R version 4.1.0, and the packages “forestplot” [24] and “survey” [25] were used. A 2-sided *p* < 0.05 was considered significant.

## 3. Results

A total of 23,758 participants were involved in this study, including 18,486 participants who reported breakfast in both recalls, 3828 participants who reported breakfast in one recall, and 1444 participants who reported breakfast in no recalls. The characteristics of participants who reported breakfast in both, one, and no recalls are shown in Table 1, and those of the 14,348 participants without obesity and 9410 participants with obesity are shown in Table S1 (Supplementary Materials). The DII was −0.36, 0.52, and 1.55 among participants who reported breakfast in both recalls, in one recall, and in no recalls, respectively, and correspondingly, their energy intakes were 2084.77 kCal, 2067.18 kCal and 1896.49 kCal, respectively.

Table 2 was the logistic regression results of dietary inflammation and eating breakfast on the obesity in three different models. In the crude model 1, pro-inflammatory diet and reported breakfast in no recalls were positively associated with obesity, and similar results were found in model 2 and final model 3. The strength of the association between the pro-inflammatory diet and skipping breakfast were stronger in the final model. Compared with the anti-inflammatory diet, the OR for the pro-inflammatory diet was 1.38 (1.22, 1.55); compared with eating breakfast in both recalls, the OR of reported breakfast in no recalls was bigger than that in one recall (1.47 (1.24, 1.75) vs. 1.18 (1.07, 1.31)).

Furthermore, we explored the mediated effect of DII on the association between eating breakfast and BMI. Figure 1 shows the three pathways linear regression among eating breakfast, DII, and BMI. Compared with the participants who reported breakfast in both recalls, the regression coefficients of reported in no recalls were higher than those of reported in one recall. The mediation analysis results shown in Table 3 indicate that compared with participants who reported breakfast in both recalls, the mediated proportions of participants who reported breakfast in one recall and in no recall were 24.71% and 27.34%, respectively.

**Figure 2:**
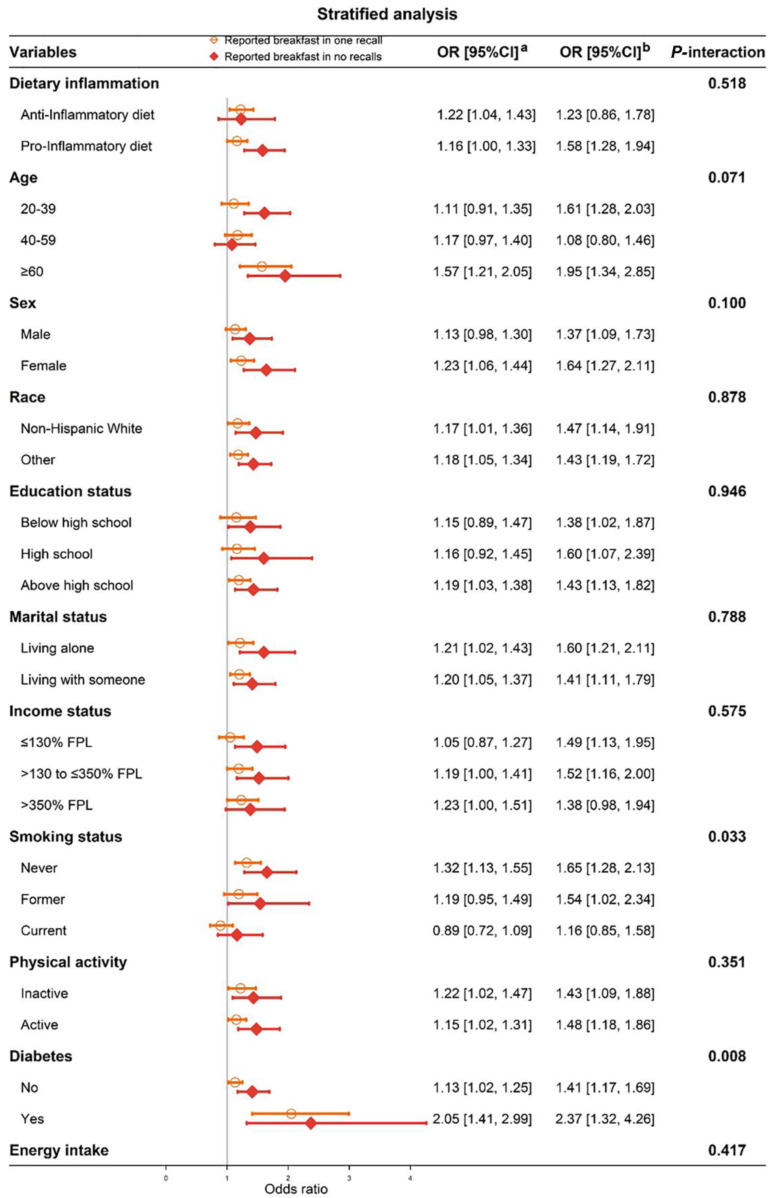
Forest plot of stratified analyses of the associations between Reported breakfast and obesity. ^a^ Reported breakfast in one recall. ^b^ Reported breakfast in no recalls.

Stratified analysis of the association between eating breakfast and obesity is shown in Figure 2 via forest plot. These associations were almost robust after sub-groups, and skipping breakfast was positively associated with obesity. The strength of this association of reported breakfast in no recall was stronger than that in one recall. Moreover, there was no interaction between eating breakfast and any variable, except for diabetes (*P*-interaction = 0.008) and smoking status (*P*-interaction = 0.033), and more details were showed in Table S2. Participants with diabetes had significantly high OR for obesity whether they reported breakfast in one recall or no recalls. Therefore, we excluded the diabetes patients from further mediated analysis, as shown in Table S3. The mediated proportions of participants who reported breakfast in one recall and in no recalls were 32.39% and 34.78%, respectively, with little change from previous results.

Moreover, we explored these associations in the elderly and non-elderly, as shown in Table S4 and S5 (Supplementary materials). The mediated proportions of DII in the non-elderly were higher (28.21% and 33.33% for reported in one recall and in no recalls, respectively) than in the whole population and correspondingly lower (20.00% and 13.68% for reporting in one recall and in no recalls, respectively) in the elderly, especially for those who reported no recalls.

## 4. Discussion

In this study, we aimed to investigate the associations among eating breakfast, DII, and BMI, and the main findings were as followed. Firstly, skipping breakfast was positively associated with DII and obesity. Secondly, the association between eating breakfast and BMI was mediated by DII, and the mediated proportions of participants who reported breakfast in one recall and in no recalls were 24.71% and 27.34%, respectively. Thirdly, diabetes patients were the sensitive population in this association. Moreover, the mediated proportion of DII was lower in the elderly and higher in the non-elderly than that in the whole population.

Some studies supported that habitually skipping breakfast was associated with elevated inflammation, including concentrations of CRP [12,26]. It was also reported that a longer fasting period with breakfast skipping increased inflammasome activity and inflammatory responses of peripheral leukocytes after lunch at later time points [11]. Our study found that skipping breakfast was associated with higher DII, which was developed to measure the inflammatory potential of diets and can be used in diverse populations to predict levels of inflammatory markers including CRP [27]. Noteworthily, the unhealthy diet was explored to play a significant role in the pathophysiology of obesity, which could be partly explained by low-grade, chronic inflammation [15,28].

A study from ANIBES indicated that the odds of abdominal obesity were higher for those who skipped breakfast when compared with those who always had breakfast [29]. A cohort study of Mexican women supported that regular breakfast might be an important dietary factor for body weight control [30]. Skipping breakfast was directly associated with overweight and obesity in an overwhelming number of studies [8]. Similarly, our study showed a positive association of skipping breakfast and obesity, and a previous study suggested that increased inflammation might be a potential linked pathway of this association [11,12]. Moreover, a previous study also indicated that prolonged fasting might lead to low-grade inflammation and impaired glucose homeostasis [11]. At present, a significant proportion of the literature on the benefits of breakfast is focused on health outcomes rather than dietary outcomes, although the two are frequently linked [7]. Therefore, it is recommended to eat breakfast regularly to reduce dietary inflammation as well as obesity.

However, what is being eaten for breakfast was also related to chronic inflammation. A previous study indicated that the consumption of an energy-dense, high-fat, fast-food–style breakfast resulted in increased postprandial oxidative stress [31]. Another study found an inverse association between higher adherence to a healthier breakfast pattern and lower odds for overweight/obesity [32]. Additionally, a 5-week, one-egg-per-day breakfast reduced the inflammatory markers tumor necrosis factor (TNF)-α and aspartate amino-transferase (AST) [33]. 

Our study showed that diabetic patients were the key population in this association, and previous studies support our finding. A meta-analysis supported that breakfast skipping was associated with a significantly increased risk of diabetes [34]. Another meta-analysis provided evidence that breakfast skipping was associated with an increased risk of T2DM, and that association was partly mediated by BMI [35]. A randomized clinical trial indicated that breakfast skipping was correlated with increased postprandial glycemic response in both healthy individuals and individuals with diabetes [36]. A potential reason might be that the consumption of breakfast is not only associated with increased satiation and appetite regulation but also with higher dietary quality in general including higher intake of fiber, vitamins, and minerals and lower intake of added sugars, which might decrease the risk of T2DM [35,37]. In addition, there was a so-called obesity paradox in the elderly population [38]; although we also found these associations of DII and obesity, the mediated proportions were lower than in the non-elderly, especially for those who reported no recalls. Previous studies found that DII was associated with BMI only in females [39] and with central obesity in postmenopausal rather than premenopausal females [40]. Thus, females might be more affected by these associations.

There are some strengths and weaknesses in the current research. Regarding the advantages, firstly, it was the first study focused on eating breakfast, DII, and obesity. Secondly, our study provides evidence for preventing obesity from a perspective of regularly eating breakfast. Thirdly, our study was based on NHANES, a nationally representative survey. Regarding the weaknesses, firstly, this was a cross-sectional study and might not have identified robust causal inferences. Secondly, the study population was located in the U.S., and the conclusions may not generalize to other populations. Thirdly, the 24 h recall diet data may have recall bias. Furthermore, we need to expand the cohort study’s sample size to explore the deep associations between skipping breakfast and obesity, specifically how dietary patterns affects the DII and how dietary inflammation affects this association.

## 5. Conclusions

The association between eating breakfast and obesity was partly mediated by DII. We recommend eating breakfast regularly to reduce dietary inflammation, as well as further obesity, especially for diabetic populations.

## Figures and Tables

**Table 1:** Characteristics of participants reporting breakfast in both, one or no recalls (N (%)).

Characteristics		Total (N = 23,758)	Reported Breakfast in Both Recalls (N = 18,486)	Reported Breakfast in One Recall (N = 3828)	Reported Breakfast in No Recalls (N = 1444)
DII (Mean (SE))		−0.10 (0.04)	−0.36 (0.03)	0.52 (0.06)	1.55 (0.09)
Dietary inflammation (N (%))					
	Anti-Inflammatory diet	11,558	9822 (58.2)	1397 (39.6)	339 (23.1)
	Pro-Inflammatory diet	12,200	8664 (41.8)	2431(60.4)	1105 (76.9)
BMI (Mean (SE))		29.15 (0.10)	29.00 (0.10)	29.53(0.18)	30.21 (0.26)
BMI group (N (%))					
	Under & healthy weight	6613	5091 (28.9)	1116 (31.3)	406 (28.4)
	Overweight	7735	6231 (33.9)	1117 (28.3)	387 (25.9)
	Obese	9410	7164 (37.2)	1595 (40.4)	651 (45.7)
Energy (Mean (SE))		2070.57 (9.87)	2084.77 (11.02)	2067.18 (21.47)	1896.49 (31.26)
Age (N (%))					
	20–39	7603	5026 (31.2)	1756 (50.4)	821 (63.1)
	40–59	8005	6341 (38.8)	1250 (34.9)	414 (28.0)
	≥60	8150	7119 (30.0)	822 (14.7)	209 (8.9)
Sex (N (%))					
	Male	11,474	8680 (46.7)	1946 (52.3)	850 (60.4)
	Female	12,284	9806 (53.3)	1882 (47.7)	606 (39.6)
Race (N (%))					
	Non-Hispanic White	10,550	8460 (69.6)	1527 (62.4)	563 (59.4)
	Other	13,208	10,026 (30.4)	2301 (37.6)	881 (40.6)
Education status (N (%))					
	Below high school	5203	4063 (13.7)	828 (15.5)	312 (16.1)
	High school	5417	4034 (21.4)	953 (25.5)	430 (33.4)
	Above high school	13,138	10,389 (64.9)	2047 (59.0)	702 (50.5)
Marital status (N (%))					
	Living alone	9485	6847 (33.6)	1832 (45.6)	806 (56.7)
	Living with someone	14,273	11,639 (66.4)	1996 (54.4)	638 (43.3)
Income status (N (%))					
	≤130% FPL	7394	5284 (18.9)	1489 (30.1)	621 (34.6)
	>130 to ≤350% FPL	9000	7097 (35.2)	1375 (33.9)	528 (35.3)
	>350% FPL	7364	6105 (45.9)	964 (36.0)	295 (30.1)
Smoking status (N (%))					
	Never	13,183	10,509 (57.7)	1962 (51.6)	712 (49.9)
	Former	5943	4938 (26.7)	793 (20.4)	212 (13.8)
	Current	4632	3039 (15.6)	1073 (28.0)	520 (36.3)
Physical activity (N (%))					
	Inactive	9397	7371 (34.6)	1476 (33.7)	550 (35.6)
	Active	14,361	11,115 (65.4)	2352 (66.3)	894 (64.4)
Diabetes (N (%))					
	No	20,475	15,743 (89.4)	3425 (92.7)	1307 (92.7)
	Yes	3283	2743 (10.6)	403 (7.3)	137 (7.3)

**Table 2:** Logistic regression model for inflammatory diet and breakfast on obesity.

	Model 1			Model 2			Model 3		
	β	*P*	OR [95%CI]	β	*P*	OR [95%CI]	β	*P*	OR [95%CI]
Dietary inflammation									
Anti-inflammatory diet	Ref	Ref	Ref	Ref	Ref	Ref	Ref	Ref	Ref
Pro-inflammatory diet	0.27	<0.001	1.31 [1.20, 1.43]	0.21	<0.001	1.23 [1.13, 1.35]	0.32	<0.001	1.38 [1.22, 1.55]
Reported breakfast									
Reported breakfast in both recalls	Ref	Ref	Ref	Ref	Ref	Ref	Ref	Ref	Ref
Reported breakfast in one recall	0.09	0.089	1.09 [0.99, 1.21]	0.14	0.006	1.15 [1.04, 1.27]	0.17	0.002	1.18 [1.07, 1.31]
Reported breakfast in no recalls	0.26	0.003	1.30 [1.10, 1.53]	0.35	<0.001	1.42 [1.19, 1.69]	0.39	<0.001	1.47 [1.24, 1.75]

Model 1 = Inflammatory diet + Reporting breakfast. Model 2 = Model 1 + Sex + Age + Race + Education status + Marital status + Income status. Model 3 = Model 2+ Smoking status + Physical activity + Diabetes + Energy intake.

**Table 3:** Mediation effect of the DII on the association between reported breakfast and BMI.

	N	Direct Effect	Mediated (Indirect) Effect	Total Effect (Exposure to Outcome)	Proportion Mediated (%)
Reported breakfast in both recalls	Ref	Ref	Ref	Ref	Ref
Reported breakfast in one recall	3828	0.64 ***	0.21 *	0.85 ***	24.71
Reported breakfast in no recalls	1444	1.01 ***	0.38 *	1.39 ***	27.34

Notes: Exposure: Reported breakfast; Outcome: BMI; Mediator: DII. Model adjusted for Sex, Age, Race, Education status, Marital status, Income status, Smoking status, Physical activity, Diabetes, and Energy intake. * *p* < 0.05; *** *p* < 0.001.

## Data Availability

Data described in the manuscript, code book, and analytic code will be made publicly and freely available without restriction at [https://www.cdc.gov/nchs/nhanes (accessed on 29 June 2022)].

## Supplementary Materials

The following supporting information can be downloaded at: https://www.mdpi.com/article/10.3390/nu14204378/s1, Figure S1: Selection process of subjects; Table S1: Characteristics of participants with and without obesity (Mean (SE)/N (%)); Table S2: Stratified regression of the effect of reported breakfast on obesity; Table S3: Sensitivity analysis results of mediation effect of the DII on the association between reported breakfast and BMI in the population without diabetes; Table S4: Sensitivity analysis results of mediated effect of the DII on the association between reported breakfast and BMI for non-elderly; Table S5: Sensitivity analysis results of mediated effect of the DII on the association between reported breakfast and BMI for elderly.

## References

[1] Caballero B. (2019). Humans against Obesity: Who Will Win?. Adv. Nutr..

[2] Polyzos S.A., Kountouras J., Mantzoros C.S. (2019). Obesity and nonalcoholic fatty liver disease: From pathophysiology to therapeutics. Metabolism.

[3] Peters U., Dixon A.E., Forno E. (2018). Obesity and asthma. J. Allergy Clin. Immunol..

[4] Silvestris E., de Pergola G., Rosania R., Loverro G. (2018). Obesity as disruptor of the female fertility. Reprod. Biol. Endocrinol..

[5] Collaborators G.B.D.O., Afshin A., Forouzanfar M.H., Reitsma M.B., Sur P., Estep K., Lee A., Marczak L., Mokdad A.H., Moradi-Lakeh M. (2017). Health Effects of Overweight and Obesity in 195 Countries over 25 Years. N. Engl. J. Med..

[6] Gibney M.J., Barr S.I., Bellisle F., Drewnowski A., Fagt S., Hopkins S., Livingstone B., Varela-Moreiras G., Moreno L., Smith J. (2018). Towards an Evidence-Based Recommendation for a Balanced Breakfast-A Proposal from the International Breakfast Research Initiative. Nutrients.

[7] Gibney M.J., Barr S.I., Bellisle F., Drewnowski A., Fagt S., Livingstone B., Masset G., Varela Moreiras G., Moreno L.A., Smith J. (2018). Breakfast in Human Nutrition: The International Breakfast Research Initiative. Nutrients.

[8] Ma X., Chen Q., Pu Y., Guo M., Jiang Z., Huang W., Long Y., Xu Y. (2020). Skipping breakfast is associated with overweight and obesity: A systematic review and meta-analysis. Obes. Res. Clin. Pract..

[9] Shimizu H., Hanzawa F., Kim D., Sun S., Laurent T., Umeki M., Ikeda S., Mochizuki S., Oda H. (2018). Delayed first active-phase meal, a breakfast-skipping model, led to increased body weight and shifted the circadian oscillation of the hepatic clock and lipid metabolism-related genes in rats fed a high-fat diet. PLoS ONE.

[10] Scheer F.A., Hilton M.F., Mantzoros C.S., Shea S.A. (2009). Adverse metabolic and cardiovascular consequences of circadian misalignment. Proc. Natl. Acad. Sci. USA.

[11] Nas A., Mirza N., Hagele F., Kahlhofer J., Keller J., Rising R., Kufer T.A., Bosy-Westphal A. (2017). Impact of breakfast skipping compared with dinner skipping on regulation of energy balance and metabolic risk. Am. J. Clin. Nutr..

[12] Zhu S., Cui L., Zhang X., Shu R., VanEvery H., Tucker K.L., Wu S., Gao X. (2021). Habitually skipping breakfast is associated with chronic inflammation: A cross-sectional study. Public Health Nutr..

[13] Shivappa N., Steck S.E., Hurley T.G., Hussey J.R., Hebert J.R. (2014). Designing and developing a literature-derived, population-based dietary inflammatory index. Public Health Nutr..

[14] Tan Q.Q., Du X.Y., Gao C.L., Xu Y. (2021). Higher Dietary Inflammatory Index Scores Increase the Risk of Diabetes Mellitus: A Meta-Analysis and Systematic Review. Front. Endocrinol..

[15] Hariharan R., Odjidja E.N., Scott D., Shivappa N., Hebert J.R., Hodge A., de Courten B. (2022). The dietary inflammatory index, obesity, type 2 diabetes, and cardiovascular risk factors and diseases. Obes. Rev..

[16] Suhett L.G., Lopes L.J., Silva M.A., Vieira Ribeiro S.A., Hermsdorff H.M., Shivappa N., Hebert J.R., Novaes J.F. (2022). Interaction effect between breakfast skipping and sedentary behavior in the dietary inflammatory potential of Brazilian school-age children. Nutrition.

[17] Haghighatdoost F., Feizi A., Esmaillzadeh A., Keshteli A.H., Afshar H., Adibi P. (2021). Breakfast skipping alone and in interaction with inflammatory based quality of diet increases the risk of higher scores of psychological problems profile in a large sample of Iranian adults. J. Nutr. Sci..

[18] U.S. Census Bureau (2008). Current Population Survey(CPS)—Definitions and Explanations.

[19] Kant A.K., Graubard B.I. (2015). Within-person comparison of eating behaviors, time of eating, and dietary intake on days with and without breakfast: NHANES 2005–2010. Am. J. Clin. Nutr..

[20] (1995). Physical status: The use and interpretation of anthropometry. Report of a WHO Expert Committee. World Health Organ. Tech. Rep. Ser..

[21] Chang H.J., Lin K.R., Lin M.T., Chang J.L. (2021). Associations Between Lifestyle Factors and Reduced Kidney Function in US Older Adults: NHANES 1999–2016. Int. J. Public Health.

[22] Feng Q., Yang Z., May M., Tsoi K.K., Ingle S., Lee E.K., Wong S.Y., Kim J.H. (2021). The role of body mass index in the association between dietary sodium intake and blood pressure: A mediation analysis with NHANES. Nutr. Metab. Cardiovasc. Dis..

[23] Lau E., Neves J.S., Ferreira-Magalhaes M., Carvalho D., Freitas P. (2019). Probiotic Ingestion, Obesity, and Metabolic-Related Disorders: Results from NHANES, 1999–2014. Nutrients.

[24] Gordon M., Lumley T. (2016). Forestplot: Advanced Forest Plot Using ‘grid’ Graphics.

[25] Lumley T. (2014). Survey: Analysis of Complex Survey Samples. J. Stat. Softw..

[26] Guinter M.A., Campbell P.T., Patel A.V., McCullough M.L. (2019). Irregularity in breakfast consumption and daily meal timing patterns in association with body weight status and inflammation. Br. J. Nutr..

[27] Shivappa N., Steck S.E., Hurley T.G., Hussey J.R., Ma Y., Ockene I.S., Tabung F., Hebert J.R. (2014). A population-based dietary inflammatory index predicts levels of C-reactive protein in the Seasonal Variation of Blood Cholesterol Study (SEASONS). Public Health Nutr..

[28] Vozarova B., Weyer C., Lindsay R.S., Pratley R.E., Bogardus C., Tataranni P.A. (2002). High white blood cell count is associated with a worsening of insulin sensitivity and predicts the development of type 2 diabetes. Diabetes.

[29] Navia B., Lopez-Sobaler A.M., Villalobos T., Aranceta-Bartrina J., Gil A., Gonzalez-Gross M., Serra-Majem L., Varela-Moreiras G., Ortega R.M. (2017). Breakfast habits and differences regarding abdominal obesity in a cross-sectional study in Spanish adults: The ANIBES study. PLoS ONE.

[30] Martinez C.F., Ortiz-Panozo E., Mattei J., Campos H., Flores-Aldana M., Lajous M. (2021). Breakfast Frequency Is Inversely Associated with Weight Gain in a Cohort of Mexican Women. J. Nutr..

[31] Devaraj S., Wang-Polagruto J., Polagruto J., Keen C.L., Jialal I. (2008). High-fat, energy-dense, fast-food-style breakfast results in an increase in oxidative stress in metabolic syndrome. Metabolism.

[32] Cacau L.T., De Miguel-Etayo P., Santaliestra-Pasías A.M., Giménez-Legarre N., Marchioni D.M., Molina-Hidalgo C., Censi L., González-Gross M., Grammatikaki E., Breidenassel C. (2021). Breakfast Dietary Pattern Is Inversely Associated with Overweight/Obesity in European Adolescents: The HELENA Study. Children.

[33] Ballesteros M.N., Valenzuela F., Robles A.E., Artalejo E., Aguilar D., Andersen C.J., Valdez H., Fernandez M.L. (2015). One Egg per Day Improves Inflammation when Compared to an Oatmeal-Based Breakfast without Increasing Other Cardiometabolic Risk Factors in Diabetic Patients. Nutrients.

[34] Bi H., Gan Y., Yang C., Chen Y., Tong X., Lu Z. (2015). Breakfast skipping and the risk of type 2 diabetes: A meta-analysis of observational studies. Public Health Nutr..

[35] Ballon A., Neuenschwander M., Schlesinger S. (2019). Breakfast Skipping Is Associated with Increased Risk of Type 2 Diabetes among Adults: A Systematic Review and Meta-Analysis of Prospective Cohort Studies. J. Nutr..

[36] Jakubowicz D., Wainstein J., Landau Z., Raz I., Ahren B., Chapnik N., Ganz T., Menaged M., Barnea M., Bar-Dayan Y. (2017). Influences of Breakfast on Clock Gene Expression and Postprandial Glycemia in Healthy Individuals and Individuals With Diabetes: A Randomized Clinical Trial. Diabetes Care.

[37] St-Onge M.P., Ard J., Baskin M.L., Chiuve S.E., Johnson H.M., Kris-Etherton P., Varady K. (2017). Meal Timing and Frequency: Implications for Cardiovascular Disease Prevention: A Scientific Statement From the American Heart Association. Circulation.

[38] Oreopoulos A., Kalantar-Zadeh K., Sharma A.M., Fonarow G.C. (2009). The obesity paradox in the elderly: Potential mechanisms and clinical implications. Clin. Geriatr. Med..

[39] Ruiz-Canela M., Zazpe I., Shivappa N., Hébert J.R., Sánchez-Tainta A., Corella D., Salas-Salvadó J., Fitó M., Lamuela-Raventós R.M., Rekondo J. (2015). Dietary inflammatory index and anthropometric measures of obesity in a population sample at high cardiovascular risk from the PREDIMED (PREvención con DIeta MEDiterránea) trial. Br. J. Nutr..

[40] Kim H.Y., Lee J., Kim J. (2018). Association between Dietary Inflammatory Index and Metabolic Syndrome in the General Korean Population. Nutrients.

