# Supplementary material for: The Mediating Role of Dietary Inflammatory Index in the Association between Eating Breakfast and Obesity: A Cross-Sectional Study
Source: Nutrients. 2022 Oct 19;14(20):4378. doi: 10.3390/nu14204378 (PMC9608633; doi:10.3390/nu14204378)
Supplement: Supplementary file 1 [file nutrients-14-04378-s001.zip › nutrients-1965846-supplementary.pdf]

Supplemental Material

The Mediating Role of Dietary Inflammatory Index in the Association between Eating Breakfast and Obesity: A Cross-Sectional Study

Mengzi Sun †, Xuhan Wang †, Ling Wang, Wenyu Hu, Yixue Yang, Nan Yao, Jing Li, Zechun Xie, Ruirui Guo, Yuxiang Wang and Bo Li *

Department of Epidemiology and Biostatistics, School of Public Health, Jilin University, Changchun 130021, China

* Correspondence: li_bo@jlu.edu.cn

† These authors contributed equally to this work.

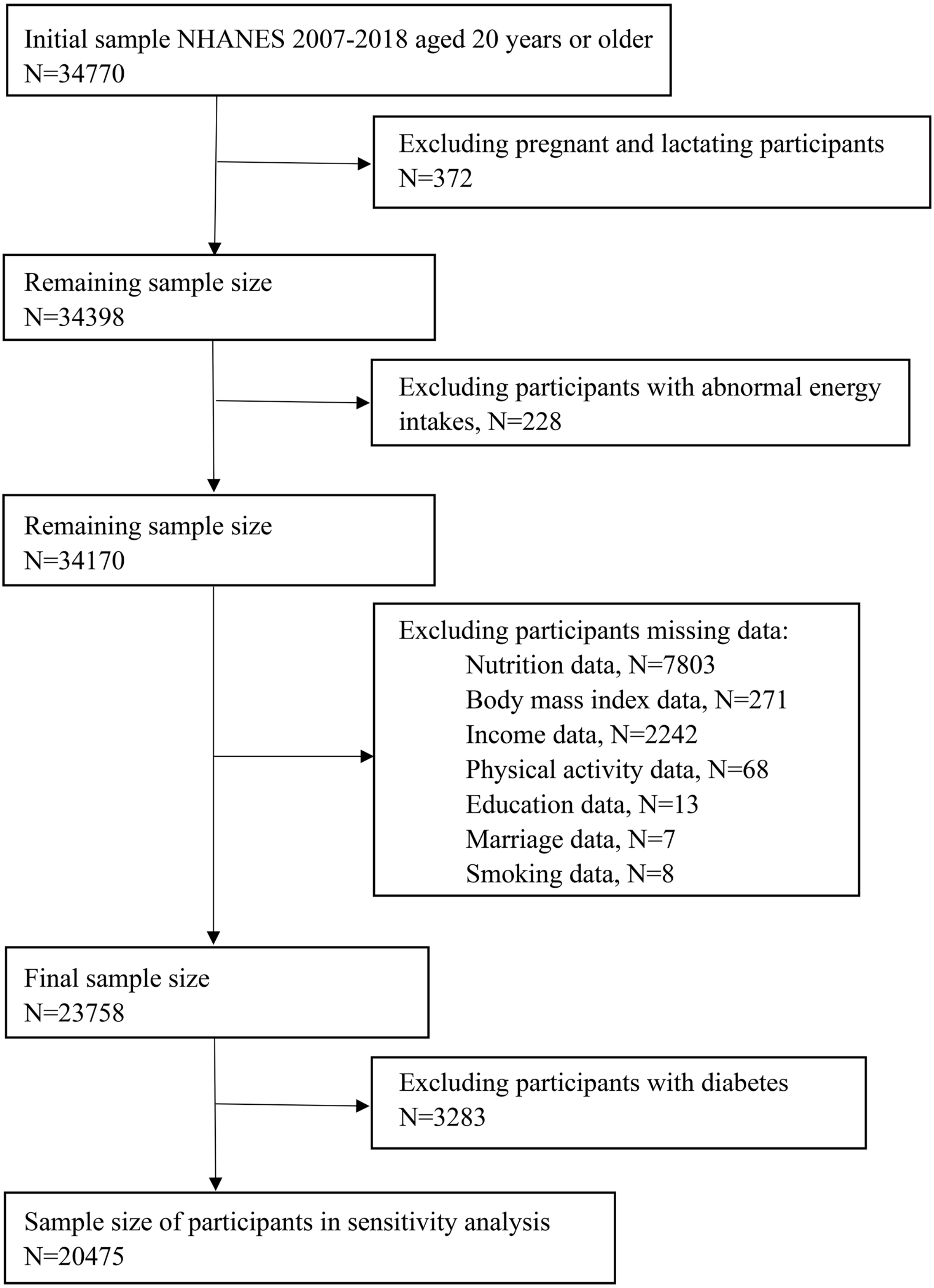

Figure S1. Selection process of subjects

Table S1. Characteristics of participants with and without obesity (Mean (SE)/N (%))

Characteristics	Non-obesity (N=14348)	Obesity (N=9410)	t/χ^2	*P*
DII (Mean (SE))	-0.24(0.04)	0.12(0.04)	8.447	<0.001
Dietary inflammation (N (%))			122.128	<0.001
Anti-Inflammatory diet	7382(55.9)	4176(48.5)		
Pro-Inflammatory diet	6966(44.1)	5234(51.5)		
Reported breakfast (N (%))			50.367	<0.001
Reported breakfast in both recalls	11322(79.1)	7164(75.7)		
Reported breakfast in one recall	2233(15.6)	1595(17.1)		
Reported breakfast in no recalls	793(5.3)	651(7.2)		
Age (N (%))			79.701	<0.001
20-39	4869(38.4)	2734(32.7)		
40-59	4567(36.4)	3438(39.5)		
≥60	4912(25.2)	3238(27.8)		
Sex (N (%))			11.152	0.054
Male	7327(49.3)	4147(47.0)		
Female	7021(50.7)	5263(53.0)		
Race (N (%))			56.849	<0.001
Non-Hispanic White	6574(69.7)	3976(65.0)		
Other	7774(30.3)	5434(35.0)		
Education status (N (%))			73.460	<0.001
Below high school	3042(13.5)	2161(15.4)		
High school	3114(21.4)	2303(25.0)		
Above high school	8192(65.1)	4946(59.6)		
Marital status (N (%))			0.350	0.679
Living alone	5666(36.8)	3819(37.2)		
Living with someone	8682(63.2)	5591(62.8)		
Income status (N (%))			63.465	<0.001
≤130% FPL	4302(20.6)	3092(23.4)		
>130 to ≤350% FPL	5307(34.1)	3693(36.5)		
>350% FPL	4739(45.3)	2625(40.1)		
Smoking status (N (%))			60.933	<0.001
Never	7961(56.8)	5222(55.5)		
Former	3383(23.3)	2560(27.3)		
Current	3004(19.9)	1628(17.2)		
Physical activity (N (%))			306.594	<0.001
Inactive	5205(30.3)	4192(41.4)		
Active	9143(69.7)	5218(58.6)		
Diabetes (N (%))			855.483	<0.001
No	13087(94.6)	7388(83.0)		
Yes	1261(5.4)	2022(17.0)		
Energy intake (Mean (SE))	2085.40(11.90)	2046.60(13.85)	-2.378	0.019

Table S2. Stratified regression of the effect of reported breakfast on obesity

Characteristics	Reported breakfast in one recall (N=3828)		Reported breakfast in no recalls (N=1444)		*P*-interaction
	P	OR [95%CI]	*P*	OR [95%CI]	
Dietary inflammation					0.518
Anti-Inflammatory diet	0.014	1.22 [1.04, 1.43]	0.260	1.23 [0.86, 1.78]	
Pro-Inflammatory diet	0.047	1.16 [1.00, 1.33]	<0.001	1.58 [1.28, 1.94]	
Age					0.071
20-39	0.292	1.11 [0.91, 1.35]	<0.001	1.61 [1.28, 2.03]	
40-59	0.094	1.17 [0.97, 1.40]	0.605	1.08 [0.80, 1.46]	
≥60	0.001	1.57 [1.21, 2.05]	0.001	1.95 [1.34, 2.85]	
Sex					0.100
Male	0.090	1.13 [0.98, 1.30]	0.007	1.37 [1.09, 1.73]	
Female	0.007	1.23 [1.06, 1.44]	<0.001	1.64 [1.27, 2.11]	
Race					0.878
Non-Hispanic White	0.035	1.17 [1.01, 1.36]	0.004	1.47 [1.14, 1.91]	
Other	0.009	1.18 [1.05, 1.34]	<0.001	1.43 [1.19, 1.72]	
Education status					0.946
Below high school	0.285	1.15 [0.89, 1.47]	0.035	1.38 [1.02, 1.87]	
High school	0.204	1.16 [0.92, 1.45]	0.021	1.60 [1.07, 2.39]	
Above high school	0.020	1.19 [1.03, 1.38]	0.004	1.43 [1.13, 1.82]	
Marital status					0.788
Living alone	0.030	1.21 [1.02, 1.43]	0.001	1.60 [1.21, 2.11]	
Living with someone	0.008	1.20 [1.05, 1.37]	0.006	1.41 [1.11, 1.79]	
Income status					0.575
≤130% FPL	0.609	1.05 [0.87, 1.27]	0.005	1.49 [1.13, 1.95]	
>130 to ≤350% FPL	0.050	1.19 [1.00, 1.41]	0.002	1.52 [1.16, 2.00]	
>350% FPL	0.053	1.23 [1.00, 1.51]	0.062	1.38 [0.98, 1.94]	
Smoking status					0.033
Never	0.001	1.32 [1.13, 1.55]	<0.001	1.65 [1.28, 2.13]	
Former	0.123	1.19 [0.95, 1.49]	0.043	1.54 [1.02, 2.34]	
Current	0.253	0.89 [0.72, 1.09]	0.346	1.16 [0.85, 1.58]	
Physical activity					0.351
Inactive	0.032	1.22 [1.02, 1.47]	0.011	1.43 [1.09, 1.88]	
Active	0.028	1.15 [1.02, 1.31]	0.001	1.48 [1.18, 1.86]	
Diabetes					0.008
No	0.026	1.13 [1.02, 1.25]	<0.001	1.41 [1.17, 1.69]	
Yes	<0.001	2.05 [1.41, 2.99]	0.004	2.37 [1.32, 4.26]	
Energy intake					0.417

Table S3. Sensitivity analysis results of mediation effect of the DII on the association between reported breakfast and BMI in the population without diabetes

	N	Direct Effect	Mediated (Indirect) Effect	Total Effect (Exposure to Outcome)	Proportion Mediated (%)
Reported breakfast in both recalls	Ref	Ref	Ref	Ref	Ref
Reported breakfast in one recall	3425	0.48***	0.23*	0.71***	32.39
Reported breakfast in no recalls	1307	0.75***	0.40*	1.15***	34.78

Notes: Exposure: Reported breakfast; Outcome: BMI; Mediator: DII.

Model adjusted for Sex, Age, Race, Education status, Marital status, Income status, Smoking status, Physical activity, and Energy intake.

*$p < 0.05$; ***$p < 0.001$.

Table S4. Sensitivity analysis results of mediated effect of the DII on the association between reported breakfast and BMI for non-elderly

	N	Direct Effect	Mediated (Indirect) Effect	Total Effect (Exposure to Outcome)	Proportion Mediated (%)
Reported breakfast in both recalls	Ref	Ref	Ref	Ref	Ref
Reported breakfast in one recall	3006	0.56***	0.22*	0.78***	28.21
Reported breakfast in no recalls	1235	0.80***	0.40*	1.20***	33.33

Notes: Exposure: Reported breakfast; Outcome: BMI; Mediator: DII.

Model adjusted for Sex, Age, Race, Education status, Marital status, Income status, Smoking status, Physical activity, Diabetes, and Energy intake.

*p < 0.05; ***p < 0.001.

Table S5. Sensitivity analysis results of mediated effect of the DII on the association between reported breakfast and BMI for elderly

	N	Direct Effect	Mediated (Indirect) Effect	Total Effect (Exposure to Outcome)	Proportion Mediated (%)
Reported breakfast in both recalls	Ref	Ref	Ref	Ref	Ref
Reported breakfast in one recall	822	0.84***	0.21*	1.05***	20.00
Reported breakfast in no recalls	209	2.02***	0.32*	2.34***	13.68

Notes: Exposure: Reported breakfast; Outcome: BMI; Mediator: DII.

Model adjusted for Sex, Age, Race, Education status, Marital status, Income status, Smoking status, Physical activity, Diabetes, and Energy intake.

*$p < 0.05$; ***$p < 0.001$.
